# What Are the Molecules Involved in Regulatory T-Cells Induction by Dendritic Cells in Cancer?

**DOI:** 10.1155/2013/806025

**Published:** 2013-05-22

**Authors:** Rodrigo Nalio Ramos, Cristiano Jacob de Moraes, Bruna Zelante, José Alexandre M. Barbuto

**Affiliations:** Department of Immunology, Institute of Biomedical Sciences, University of Sao Paulo, Avenida Professor Lineu Prestes, 1730 Sao Paulo, SP, Brazil

## Abstract

Dendritic cells (DCs) are essential for the maintenance of homeostasis in the organism, and they do that by modulating lymphocyte priming, expansion, and response patterns according to signals they receive from the environment. The induction of suppressive lymphocytes by DCs is essential to hinder the development of autoimmune diseases but can be reverted against homeostasis when in the context of neoplasia. In this setting, the induction of suppressive or regulatory T cells contributes to the establishment of a state of tolerance towards the tumor, allowing it to grow unchecked by an otherwise functional immune system. Besides affecting its local environment, tumor also has been described as potent sources of anti-inflammatory/suppressive factors, which may act systemically, generating defects in the differentiation and maturation of immune cells, far beyond the immediate vicinity of the tumor mass. Cytokines, as IL-10 and TGF-beta, as well as cell surface molecules like PD-L1 and ICOS seem to be significantly involved in the redirection of DCs towards tolerance induction, and recent data suggest that tumor cells may, indeed, modulate distinct DCs subpopulations through the involvement of these molecules. It is to be expected that the identification of such molecules should provide molecular targets for more effective immunotherapeutic approaches to cancer.

## 1. Background

Regulatory T cells (Tregs) are crucial to the maintenance of tolerance to autoantigens [1]. The failure of Treg function or their depletion has been implicated in the development of many autoimmune diseases in humans and in mouse models [2]. However, Treg-mediated suppressive activity can also contribute to the immune escape of pathogens or tumors [3, 4]. Nowadays, regulatory T cells (Tregs) are considered one of the major obstacles to the success of immunotherapeutic approaches to cancer [5–8]. Several studies have described the direct association between Treg increase and tumor development, implicating this phenomenon as one of the most important escape mechanisms in different tumor types [7, 9, 10]. Many evidences have demonstrated that Treg accumulation is not restricted to the tumor site but is observed in the peripheral blood as well, from patients with distinct malignant tumors, including pancreas and breast [11], lung [12], and ovarian cancer [4, 12]. Indeed, elimination of Tregs in mouse tumor models can improve antitumor immune responses and survival [9, 13].

Dendritic cells (DCs) are believed to act as sensors of the homeostatic equilibrium of their environment, where they capture antigens to present to T lymphocytes. Thus, depending on the status of the tissue, they might induce immunity or tolerance to the antigens they present. Indeed, many *in vitro* studies have demonstrated that DCs are essential for regulatory T-cells induction [14, 15], apparently depending on various distinct mechanisms [16], but also, frequently, on external sources of cytokines, among which TGF-beta seems to play a predominant role [17]. Not surprisingly, therefore, during tumor development the balancing role of DCs in the T helper versus Treg stimulation seems to be deeply modified [8, 18]. 

However, despite all the accumulated data, the precise role of DCs in the imbalance between T helper and Tregs in cancer is still unclear. Do the observed biases of DC function in tumor bearers reflect a previous disturbance in their immune homeostasis or are these deviations of DC function the cause of the other immunological abnormalities? How significant is the contribution of these DC deficits to the escape of tumors from the body's control? Though the answer to these questions is not available yet, the increasing knowledge and characterization of DC behavior in the presence of tumors allows us to predict that it will be, and, furthermore, that, once reached, it will provide us with powerful tools for the clinical management of cancer. With these goals in view, we discuss, here, the impact of tumor presence in the membrane phenotype and function of DCs and their bias to induce/expand regulatory T cells.

## 2. The Tumor Microenvironment: A Tolerogenic Milieu

Several studies have described the potential impact of tumor-derived products in the suppression of immunity. Signals derived from tumors not only act directly upon immune effector cells but also induce the conversion and/or the recruitment of cells with suppressive functions to their microenvironment [19]. In consequence, tumors are typically characterized by the presence of higher concentrations of anti-inflammatory molecules, such as TGF-beta, IL-10, and prostaglandin E2 [20–23], increased amounts of angiogenic factors, as the vascular endothelial growth factor (VEGF) [24], and augmented CCL22 chemokine gradient [25] in addition to the local expression of immune-inhibitory molecules, including CTLA4 and PD-1/PD-L1 [26, 27]. Altogether, these constitute, nowadays, the most highly sought targets to achieve the breakdown of tumor-associated microenvironment-induced tolerance. Still, in order to obtain an immune recovery in face of tumors, we still need to identify the source of the tolerogenic signals. Though tumors cells may produce such mediators, also tumor-infiltrating leukocytes may be their source, and, indeed, the study of such populations has revealed that regulatory Foxp3^+^ T cells (Tregs) [28], anti-inflammatory M2-macrophages [29], plasmacytoid dendritic cells (pDCs) [30], and immature myeloid DCs [31] accumulate in human neoplastic tissues and patients' blood [4] and have been associated with poor prognosis for the patients specific cancer types. 

As mentioned, the presence of tolerance-inducing conditions seems not to be restricted to the tumor microenvironment. Several studies have demonstrated the increase of anti-inflammatory cytokines and the higher frequency of suppressive cells in the bloodstream and lymph nodes from cancer patients. The detection of higher amounts of cytokines like TGF-beta [32], M-CSF [33], and IL-6 [34, 35] in patients' serum could suggest that the tumor presence affects cells in distant organs, thus resulting in systemic alterations which could allow tumors not only to grow locally unchecked but also to metastasize without an effective immune barrier. In agreement with that are: the higher frequency of myeloid-derived suppressor cells (MDSCs) (a group of immature but potent suppressor cells capable of down-regulating anti-tumor immunity) found in cancer patients' circulation [36]; the decreased frequency of circulating and tumor-infiltrating myeloid DCs [37, 38]; and the CD4 lymphopenia observed in cancer patients [39–41]; all three important alterations of immune homeostasis in cancer patients that, consequently, hamper the effectiveness of their treatment.

## 3. DCs: Targets to the Tumor Tolerogenic Milieu 

Dendritic cells (DCs) are the best adapted professional antigen-presenting cells (APCs) able to initiate, coordinate, and regulate the adaptive immune responses by inducing naive T-cells differentiation into diverse T helper lymphocyte subtypes [42–46]. Generally, at homeostasis condition, tissue-resting DCs are in immature status (lower MHC class II and costimulatory molecules expression) and strategically located to sense and acquire antigenic products from the environment. Using nonspecific receptors, immature DCs can recognize pathogens or danger-associated molecular patterns (as known, PAMPs and DAMPs, resp.) and migrate to lymphoid organs, at the same time as they increase their expression of MHC, CD80, CD86, and CD40 surface molecules and become ready to activate naïve T lymphocytes [44]. DCs are also crucial for the induction/maintenance of T-cell tolerance to antigens acquired in “healthy” tissues, thus performing an essential role in the prevention of autoimmunity [47].

It is also evident that the term DC is applied to several distinct subpopulations, classified, still incompletely, in relation to their tissue localization, migratory ability, surface markers' expression, and the profile of soluble factors they release. Though still uncertain, it is becoming increasingly clear that any classification of DCs will be insufficient to accommodate all the plasticity of these cells. Therefore, a better approach to the problem would be to describe, as well as possible, the DCs found in a certain condition, and from that, to correlate their phenotype in that specific situation with the known functions of these cells. This has been done in relation to DCs within tumors and has shown that tumors modify significantly the phenotype of DCs within their microenvironment [8, 22]. Various observations point to a mainly functional deficit of these cells in immune stimulation, due to a decreased frequency of mature, functionally competent DCs within tumors [31] and in peripheral blood [48]. Actually, we have already shown an altered expression of CD86 in Mo-DCs from advanced cancer patients, which was, apparently, corrected by an immunotherapeutic approach [49]. Importantly, the presence of pDCs in tumor sites has been also related to poor prognosis in cancer patients [30], and their functional investigation revealed a considerable low to absent IFN-alpha production in breast and ovarian cancer [50, 51]. The tumor-associated stroma and cancer cells *per se* can generate signals that drive DC to a tolerogenic pathway, characterized, mainly, by a poor upregulation of MHC class II and costimulatory molecules and absent or low production of proinflammatory cytokines [52], thus favoring tumor evasion from the immune system. Interestingly, in tumor-bearing mouse, the presence of DCs is also crucial for cancer vascularization, and when DCs are depleted, the elimination of malignant cells can be enhanced [53, 54]. Additionally, another elegant study showed that human myeloid DCs expressing OX40L stimulate Th2 immunity *in vitro,* under the influence of thymic stromal lymphopoietin (TSLP) derived from breast tumor cells [55]. Such findings may explain the bias towards a Th2 inflammatory tumor microenvironment found in breast cancer. 

Since it became possible to achieve DC differentiation from human blood monocytes (Mo-DCs) [56], the immunostimulatory potential of these cells could be harnessed for cancer immunotherapy [57–60]. On the other hand, *in vitro* findings, describe that tumor cells present during human monocyte differentiation cause alteration in their molecular expression and unsuccessful DC differentiation, even under exogenous cytokine addition [61–63]. In addition, we have shown that breast cancer patients' monocyte-derived DCs are phenotypic altered and biased to induce Tregs [64], even though differentiated without the presence of tumor cells in the culture. 

**Figure 1:**
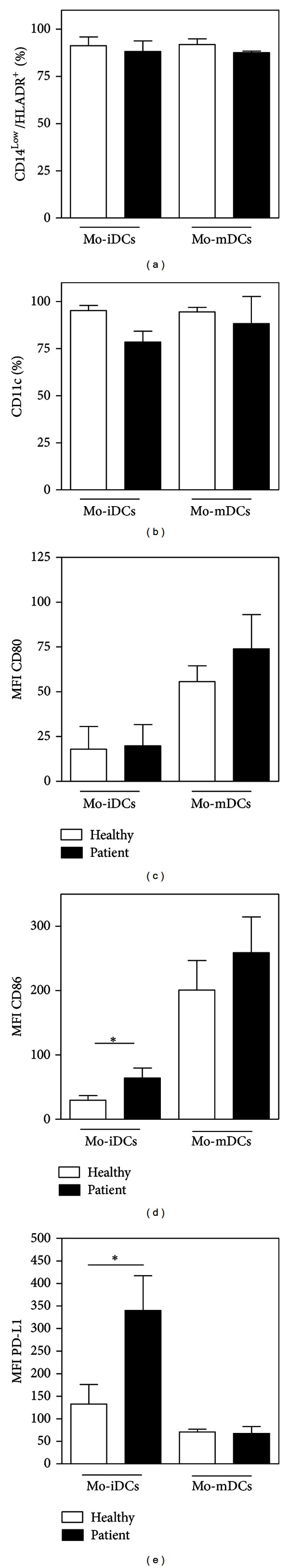
Patients' immature Mo-DCs express higher levels of PD-L1. Blood monocytes from control, and breast cancer patient subjects were cultured in the presence of IL-4 and GM-CSF for seven days and, subsequently, characterized. Flow cytometry analysis showing grouped frequency of CD14^Low^/HLADR^+^ (a) and CD11c^+^ (b) cells and Mean Intensity Fluorescence (MFI) values of CD14^Low^/HLADR^+^ gated cells to CD80 (c), CD86 (d), and PD-L1 (e) molecules in Mo-iDCs from healthy donors and breast cancer patients (*P < 0.05, two-tailed unpaired *t-*test; healthy *n* = 5; patients *n* = 9). (Mature Mo-DCs were activated by TNF-alfa for 48 hours.)

Immature Mo-DCs from patients express higher levels of CD86 and PD-L1 membrane molecules after 7 days in the presence of IL-4 and GM-CSF (Figure 1). Though the expression of CD86 could be interpreted as an enhanced costimulatory ability, the same cannot be implied for PD-L1. PD-L1, also known as B7-H1, has been described as an inhibitory molecule in T lymphocyte activation [65, 66] and also related to T effector to Treg conversion [67] and the induction of T cell anergy by Mo-DCs [68]. Furthermore, its expression has been described as enhanced in monocytes from peritumoral stroma in hepatocellular carcinoma [26] and in lung cancer infiltrating DCs [69]. 

## 4. Regulatory T-Cells Induction by Tumor-Affected DCs 

**Figure 2:**
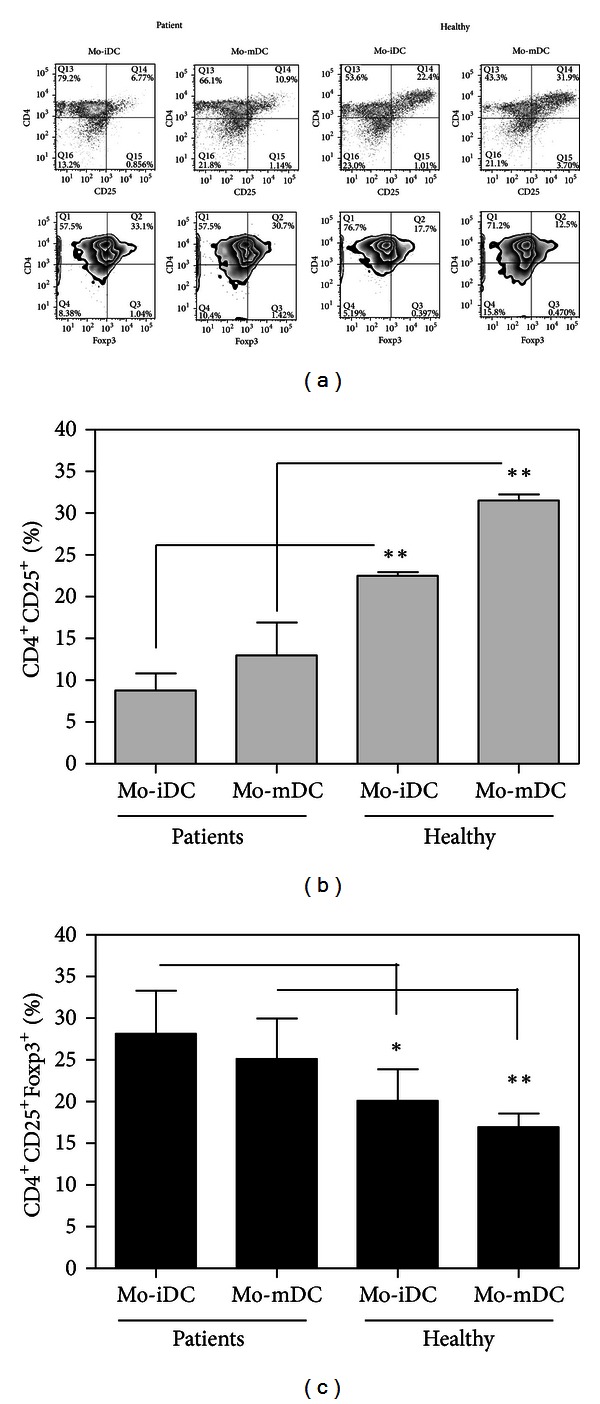
Patients' Mo-DCs fail to activate CD4^+^ lymphocytes and induce higher Foxp3 expression even after maturation. Mo-DCs from controls and breast cancer patients were cocultured with allogeneic CD4^+^CD45RA^+^ cells for five days. At the end of culture the phenotype of lymphocytes was evaluated by flow cytometry. (a) Representative experiments of CD25 and Foxp3 expression in CD4^+^ lymphocytes stimulated by immature DCs (Mo-iDCs) or mature DCs (Mo-mDCs) from healthy donors or breast cancer patients. Average frequency of CD25^+^ cells (b) and CD4^+^CD25^+^Foxp3^+^ cells (c) after CD4^+^CD45RA^+^ lymphocytes' coculture with Mo-DCs (*P < 0.05;  **P < 0.01, two-tailed unpaired *t-*test; *n* = 4). (Mature Mo-DCs were activated by TNF-alfa for 48 hours.)

The induction and expansion of Tregs by DCs are generally related to their role in the maintenance of tolerance to self [16]. Several studies have been developed, trying to identify the signals that drive DCs into that function and, thus, eventually allow the use of such educated DCs to control unwanted immune responses, like those against transplanted tissues or in autoimmune diseases [70]. Actually, the acquisition of the ability to promote Tregs is an integral part of the physiologic function of DCs, as can be noted, for instance, in the presence of apoptotic cells [71, 72]. In this search, anti-inflammatory cytokines as IL-10 [73, 74], TGF-beta [75], and vitamin D3 addition [76, 77] have been shown to affect mouse and human DCs, causing them to stimulate regulatory or suppressive T lymphocytes [78]. Intriguingly, even inflammatory cytokines, as TNF-alpha, have been associated with tolerogenic DC induction in autoimmune disorders like the murine Experimental Autoimmune Encephalomyelitis (EAE) [79]. Paradoxically, the same functional status of DCs, which is the still unreached aim of research in autoimmunity and transplantation studies, is the natural status of DCs in cancer, which is, again, beyond our powers of effective modulation. Tumor cells are associated with lower activation of immune cells and hinder APC activation [32, 80, 81] and, also can attract regulatory T cells to their microenvironment [4, 11, 12], all phenomena which would be more than welcome in the aforementioned autoimmune and transplant recipients. Regarding APCs, *in vitro* studies showed Treg induction by human Mo-DCs stimulated by pancreatic or lung tumor cells [61, 62], the ability of human intratumoral pDCs, to expand Tregs *ex vivo* in breast cancer [50] and to induce suppressive activity by T cells in prostate cancer [82]. These findings show that tumor cells are able to promote Tregs induction by DCs in patients, and also to affect DCs from healthy donors, causing them to stimulate Tregs. Finally, our group has demonstrated that this effect of tumors upon DCs does not depend on the continuous presence of neoplastic cells, since Mo-DCs from breast cancer patients even when differentiated *in vitro* and, therefore, away from the direct tumor influence, are poor T-cell stimulators and biased to induce CD4^+^CD25^+^Foxp3^+^ regulatory T cells when cocultured with naïve CD4^+^CD45RA^+^ lymphocytes (Figure 2). It should be noted that this bias was present, regardless of the maturation stimulus used to activate the patients' Mo-DCs [64]. Taken together, these data indicate that during tumor development a systemic tolerogenic status of DCs is favored, enhancing their ability to expand/recruit Tregs and whose specific mechanisms are still largely undetermined.

## 5. Potential Mechanisms of Tumor-Affected DCs in the Induction of Tregs

**Figure 3:**
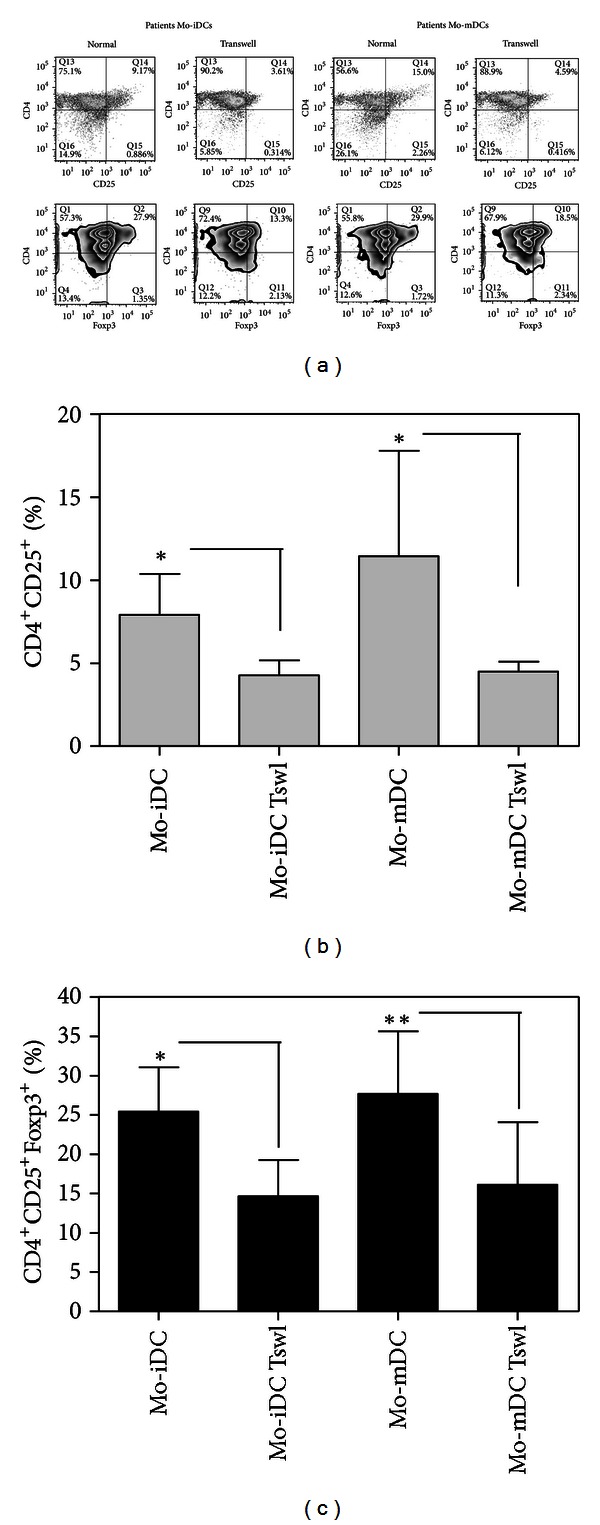
Patients' Mo-DCs induce expansion of regulatory T lymphocytes with the cooperation of contact molecules. Mo-DCs from breast cancer patients were cocultured with allogeneic CD4^+^CD45RA^+^ cells for five days in a transwell system or not. At the end of culture the phenotype of lymphocytes was evaluated by flow cytometry. (a) Representative experiments of CD25 and Foxp3 expression in CD4^+^ lymphocytes stimulated by immature DCs (Mo-iDCs) or mature DCs (Mo-mDCs) from breast cancer patients in normal or transwell condition. Average frequency of CD25^+^ cells (b) and CD4^+^CD25^+^Foxp3^+^ cells (c) after CD4^+^CD45RA^+^ lymphocytes' coculture with patients' Mo-DCs (*P < 0.05;  **P < 0.01, paired *t-*test; *n* = 4). (Tswl: transwell system; mature Mo-DCs were activated by TNF-alfa for 48 hours.)

Cytokines, as TGF-beta and IL-10 in addition to IL-2, are currently used to expand effectively murine and human Tregs *in vitro*. Interestingly, the same cytokines can also induce DCs to stimulate Tregs *in vitro* [17, 74, 75]. This may suggest that the major signals responsible for the generation and expansion of regulatory T cells *in vitro* and in vivo are already known. However, few data are available in regards to the mechanism of tumor-conditioned DCs in Tregs induction. Recent findings have demonstrated that infiltrating pDCs from ovarian [83] and breast tumor [84] can express high levels of ICOS-L, a phenomenon that could explain their ability to stimulate Foxp3^+^ Tregs *in vitro*. Our own data also have shown that the Tregs induction by Mo-DCs from cancer patients could be partially reversed by blocking of TGF-beta *in vitro*, and not by LPS, proinflammatory cocktail, or sCD40L activation [64]. TGF-beta is a multifunctional cytokine that regulates T-cell growth and development [85], inhibits IL-2 production, and has potent antiproliferative effects on CD4^+^ T cells [86], principally by inducing regulatory T cells [87]. However, since blocking of this cytokine was not enough to abolish the Treg-induction bias of the patients' Mo-DCs, it is likely that the TGF-beta signal may act together with other factors. Among the candidates for this cosignaling it is interesting to note that patients' Mo-DCs expressed higher levels of surface CD86 and PD-L1 (Figure 1), both molecules that have been also implicated in the balance of Tregs stimulation [88–91]. Thus, the TGF-beta signal may actuate together with surface molecules signals to “complement” the patients' Mo-DCs signalization in the induction/expansion of Tregs, as we showed here that DC-T cell contact is essential in that phenomenon (Figure 3).

## 6. Concluding Remarks

Tregs are recognized as central in the maintenance of tolerance to self [1] but may be also involved in the failure of the immune system to eliminate or control infections [3], tumors [13] and to respond to therapeutic vaccination [92]. Nowadays, it is also broadly accepted that DCs may play a crucial role in tolerance by the induction of Tregs at peripheral tissues and organs [16]. On the other hand, it is also known that tumor cells can alter profoundly the ability of DCs to instruct the immune system to generate adaptive antitumor responses [22], thus deviating the response to tolerance. The physiological DC ability to induce Treg activation depends on various cytokines and costimulatory molecules, but the exact balance between these, particularly, in DCs from cancer patients, is still unclear. CD86 and CD80 bind to both stimulatory (CD28) and inhibitory (CTLA-4) receptors on T cells, with different affinities [93]. In human DCs, the induction and upregulation of CD86 was shown to influence significantly T-cell activation [94], while studies in knockout mice have indicated that DCs ability to generate/expand Treg subsets can be related to the balance of CD80 and CD86 [89, 95].

Confirming the significant role of CTLA-4 signaling in the immunosuppression of cancer patients, the blockage of this molecule in clinical settings by monoclonal antibodies has been able to improve significantly the survival of metastatic melanoma patients [96, 97]. Additionally, PD-L1, ICOS-L, and TGF-beta seem to emerge as good candidates for the *in vitro* manipulation of DC phenotype/function for immunotherapeutic approaches. More recently, clinical trials targeting the PD-1/PD-L1 axis with anti-PD1 monoclonal antibodies revealed their safety [98] and achieved promising results, with tumor regressions in patients with advanced cancer [99, 100], thus indicating another possible pathway to be explored in the clinic. 

Nevertheless, these data are still sparse and much needs to be determined before an effective manipulation of DC phenotype and function is achieved. In order to accomplish this, however, studies addressing the intracellular signaling pathways in tumor-affected DCs are urgently needed and may shed light on the precise mechanisms of their response to tumors as well as provide molecular targets for their effective manipulation.

## Abbreviations

APCs: Antigen-presenting cells
DCs: Dendritic cells
GM-CSF: Granulocyte macrophage colony stimulating factor
IFN-gamma: Interferon-gamma
MFI: Median fluorescence intensity
MHC: Major histocompatibility complex
Mo-DCs: Monocyte-derived dendritic cells
Mo-iDCs: Monocyte-derived immature dendritic cells
Mo-mDCs: Monocyte-derived mature dendritic cells
PBMCs: Peripheral blood mononuclear cells
pDCs: Plasmacytoid dendritic cells
TGF-beta: Transforming growth factor-beta
TNF-alpha: Tumor necrosis factor-alpha
Tregs: Regulatory T cells
TSLP: Thymic stromal lymphopoietin
Tswl: Transwell coculture system.
